# Preparation of a Stable Nanoscale Manganese Residue-Derived FeS@Starch-Derived Carbon Composite for the Adsorption of Safranine T

**DOI:** 10.3390/nano9060839

**Published:** 2019-06-01

**Authors:** Huayu Hu, Caiqiong Lin, Yanjuan Zhang, Xiunan Cai, Zuqiang Huang, Congjin Chen, Yuben Qin, Jing Liang

**Affiliations:** School of Chemistry and Chemical Engineering, Guangxi University, Nanning 530004, China; yuhuahu@163.com (H.H.); lincaiqiong168@163.com (C.L.); 15507881660@163.com (X.C.); chencongjin@gxu.edu.cn (C.C.); 13367612677@189.cn (Y.Q.); liangl6@gxu.edu.cn (J.L.)

**Keywords:** FeS nanoparticles, carbon, starch, manganese residue, adsorption, stable adsorbent

## Abstract

To develop a novel, low-cost adsorbent with natural material and industrial waste as raw materials, nanoscale manganese residue-derived FeS@starch-derived carbon (MR–FeS@SC) composite was prepared by the carbonization of starch–manganese residue gel. Manganese residue-derived FeS (MR–FeS) and starch-derived carbon (SC) were also prepared as contrasts for comparative studies. The MR–FeS@SC nanocomposite exhibited relatively large specific surface area and micropore volume, appropriate pore size, abundant functional groups, strong interaction between the functional groups of SC and MR–FeS, and the immobilization and uniform distribution of MR–FeS nanoparticles onto SC support material, which contributed to better adsorption properties for the removal of Safranine T (ST) from the aqueous solution compared with those of MR–FeS and SC. The adsorption could be conducted at a wide range of pH and temperature to achieve a satisfy removal efficiency of ST with MR–FeS@SC nanocomposite as adsorbent. The adsorption kinetics well followed the pseudo-second-order model, and the dominant mechanism was chemisorption. The adsorption behavior was well described by the Langmuir isotherm model. Due to the strong interaction between MR–FeS nanoparticles and SC support, MR–FeS@SC nanocomposite exhibited better reusability and stability even after fifteen cycles. This study provides a facile method of preparing effective and stable adsorbents for the treatment of dye wastewater.

## 1. Introduction

With the fast development of the industries, including textile, leather tanning, papermaking, plastic product manufacturing, coating, cosmetics, food processing, etc., synthetic dyestuffs are extensively used to color the products [1]. Due to the increasing use of dyes, it is an environmental threat as the continuing production and discharge of dye effluents to the environment [2]. As most of the dyestuffs are extremely toxic and harmful to ecosystem and human health, it is crucial to treat the industrial dye-bearing effluents before discharge, and stricter legislations on the removal of dyes from effluents have been introduced by many governmental organisations [3].

The conventional methods for the removal of these non-biodegradable dyestuffs from wastewaters include chemical coagulation, electrocoagulation/flotation process, oxidation or ozonation, photocatalysis, membrane separation, adsorption, etc. [2,4,5]. Amongst these techniques, adsorption is the most widely used procedure for effective removal of dyes from aqueous media [6]. Various materials are used as adsorbents for this purpose, such as alumina, silica gel, zeolites, natural biobased materials, porous carbon, etc. [6,7,8,9]. Low-cost adsorbents with using natural materials or the wastes/by-products generated from industries as raw materials have been developed as an alternative for current expensive adsorbents [8,10,11]. FeS, a ubiquitous and easily synthesized nontoxic mineral, has been successfully applied for treating heavy metals, arsenic, selenium, inorganic and organic contaminants, etc. [12,13]. As nanoscale materials exhibit great potential for more effective treatment of the contaminants due to the properties including small particle size, large surface area, and high reactivity, the preparation of nanosized FeS is a significant study for enhancing its application performances [13,14]. However, bare FeS nanoparticles prepared by traditional methods can rapidly agglomerate to form macroparticles due to the interactions between nanoparticles, and they are also easily oxidized to decrease the reactivity [15]. The modification of bare nanosized FeS, such as loading onto support materials or attaching proper stabilizers, can prevent the particle aggregation and thus obtain stable and dispersible FeS nanoparticles. Among various modification techniques, porous materials are considered as excellent supports for the dispersion of nanoparticles, especially porous biochar with abundant oxygen-containing functional groups prepared from the carbonization of natural organic materials (glucose, sucrose, cellulose, starch, agricultural wastes, etc.) [16,17].

Commonly, most of manganese products are produced by hydrometallurgy through acid leaching of pyrolusite with pyrites as a reductant, leaving huge quantities of solid fine leaching residue as waste. Innocent treatment is needed to eliminate the contamination of this waste residue to the local environment and ecological system [18]. At present, this residue cannot be effectively used, which brings about a series of problems, including occupying land, polluting environment, and increasing production cost of enterprises [19]. It is of great significance to develop feasible technologies for direct conversion of the residue into functional materials without purification. The compositions of this manganese residue are mainly Fe, S, Si, and O, which can be used to prepare functional materials, especially the Fe and S for the preparation of FeS. The use of appropriate support materials during the formation process of FeS can contribute to uniform dispersion of these particles to obtain nanosized FeS composites [14]. Porous carbon materials with high specific surface area have been demonstrated to be excellent support materials for immobilizing FeS nanoparticles. Starch is widely considered as a green, inexpensive, and renewable natural polymer for using as precursor to prepare the porous carbon materials [20,21,22]. Starch can dissolve and swell in hot water to prepare gel, which can disperse and immobilize the residue. During the carbonization process of starch gel for preparing carbon, FeS nanoparticles can be simultaneously formed and immobilized on the carbon support material.

Herein, a facile method for the preparation of nanoscale manganese residue-derived FeS@starch-derived carbon (MR–FeS@SC) composite as an effective adsorbent was proposed. Safranine T (ST), a typical kind of cationic azine dye, is one of the most commonly used organic dyes. ST is widely used as textile colorant for dyeing tannin, cotton, bast fibers, wool, silk, leather, and paper [23,24]. Therefore, ST was chosen to study the adsorption properties of MR–FeS@SC nanocomposite. The removal efficiency of ST from aqueous solution adsorbed by MR–FeS@SC nanocomposite and the corresponding adsorption capacity, kinetics, and equilibrium isotherms were systematically investigated with manganese residue-derived FeS (MR–FeS) and starch-derived carbon (SC) as contrasts. The reusability of the MR–FeS@SC nanocomposite adsorbent was also investigated compared with that of MR–FeS.

## 2. Materials and Methods

### 2.1. Materials

**Figure 1:**
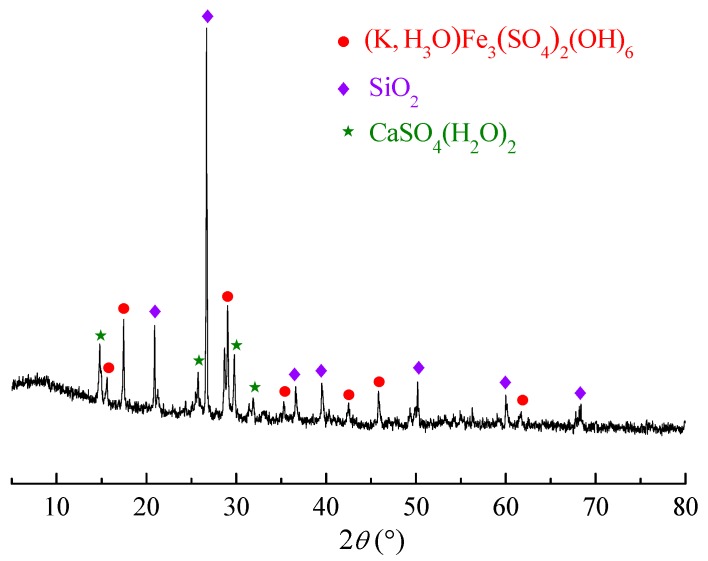
X-ray diffraction (XRD) patterns of the manganese residue.

Manganese residue was provided by Qinzhou Xiangda Chemical Co., Ltd. (Guangxi, China). The chemical compositions of the manganese residue are listed in Table 1, and Figure 1 presents the main mineralogical structures and phases, including compound sulfate ((K, H_3_O)Fe_3_(SO_4_)_2_(OH)_6_), SiO_2_, and CaSO_4_(H_2_O)_2_. Cassava starch was supplied by Guangxi State Farms Mingyang Biochemical Group, INC (Nanning, China). Safranine T was purchased from Tianjin Kemiou Chemical Reagent Co., Ltd. (Tianjin, China). All other chemical reagents (analytical grade) were obtained from commercial sources and used without further purification. Deionized water was used throughout the experiments.

### 2.2. Preparation of MR–FeS@SC Nanocomposite, MR–FeS, and SC

For the preparation of MR–FeS@SC nanocomposite, 4 g of starch and 16 g of manganese residue were mixed in a 250-mL beaker, and then 100 mL of deionized water was added to the beaker and the mixture was fully stirred at room temperature. The beaker was then put in a thermostatic water bath for heating and stirring at 80 °C for 1 h to prepare hydrogel. After cooling to room temperature, it was frozen in a refrigerator for 12 h to fix the structure of starch–manganese residue gel, which was then freeze-dried for 48 h. The dried gel was fully impregnated in 2.5 wt. % p-toluenesulfonic acid ethanol solution to stabilize its network structure and catalyze the rapid carbonization of starch [25,26]. The starch–manganese residue gel was carbonized in a tube furnace under nitrogen atmosphere at 600 °C with heating rate of 10 °C/min and holding time of 2 h. Finally, the MR–FeS@SC nanocomposite was obtained after natural cooling to room temperature under nitrogen atmosphere.

The preparation of MR–FeS (16 g of manganese residue in a batch) was operated by the same method as the preparation of MR–FeS@SC nanocomposite, without the addition of starch.

The preparation of SC (4 g of starch in a batch) was operated by the same method as the preparation of MR–FeS@SC nanocomposite, without the addition of manganese residue.

### 2.3. Characterization

The bulk chemical composition of the manganese residue was measured by an energy dispersive X-ray fluorescence (XRF) spectrometer Model (Axios, The Netherlands). X-ray diffraction (XRD) analysis was conducted on a D/MAX2500 V diffractometer (Rigaku, Japan) using Cu–Kα radiation (*λ* = 0.154 nm) at 40 kV and 30 mA, with the patterns recorded from 5° to 80°. The surface morphologies of the samples were analyzed by Field emission scanning electron microscopy (FESEM, SUPRA 55 Sapphire, Carl Zeiss, Oberkochen, Germany). Specific surface area and pore size distributions were measured by N_2_ adsorption using an ASAP 2020 detecting instrument (Micromeritics Instrument Corporation, Norcross, GA, USA) and calculated by the Brunauer–Emmett–Teller (BET) method and the Barrett–Joyner–Halenda (BJH) model, respectively [27,28]. Fourier-transform infrared (FTIR) spectra were recorded by a Nicolet IS10 spectrometer (Thermo Fisher Scientific, Waltham, MA, USA) with a resolution of 4 cm^−1^ and a wavenumber range of 400 to 4000 cm^−1^. The stoichiometric composition and chemical states were examined by X-ray photoelectron spectroscopy (XPS) on a K-Alpha^+^ X-ray photoelectron spectrometer (Thermo Fisher Scientific, USA) with a monochromatic Al Kα radiation (*hν* = 1486.6 eV). The monochromatized Al Kα X-ray source was operated in constant analyzer energy mode, and all the XPS spectra were corrected according to the C 1s line at 284.6 eV. Magnetic properties were measured by a PPMS-9 vibrating sample magnetometer (VSM, Quantum Design, Beijing, China), and the hysteretic loops were obtained under an applied magnetic field between −20,000 to 20,000 Oe at 300 K.

### 2.4. Adsorption Experiments

The adsorption of dye was performed by shaking 0.04 g of the adsorbents (0.8 g L^−1^) in 50 mL of ST solution at 130 rpm in a constant temperature shaker bath. The pH of ST solution changed between 3.0 and 11.0 was investigated using 0.1 mol L^−1^ NaOH or HCl solution. After adsorption, the MR–FeS@SC or MR–FeS adsorbent was magnetically separated by a magnet (SC was separated by centrifugation), and the solution was filtered using a 0.22 μm microporous membrane. The concentration of remnant ST in the solution was analyzed by measuring the absorbance of ST at λ_max_ = 553 nm by a UV-2802S spectrophotometer (UNICO, Shanghai, China). The removal efficiency of ST (*η*, %) and equilibrium adsorption capacity of the adsorbents (*q*_e_, mg g^−1^) were calculated using the following equations.

(1) $$\eta =\frac{C_{0}-C_{e}}{C_{0}}\times 100\%$$

(2) $$q_{e}=\frac{V\left(C_{0}-C_{e}\right)}{m}$$

where *C*_0_ and *C*_e_ (mg L^−1^) are the concentrations of the ST solution at the initial stage and under equilibrium conditions, respectively, *V* (L) is the volume of the ST solution, and *m* (g) is the mass of the adsorbent.

### 2.5. Recyclability Experiments

The recyclability of both MR–FeS and MR–FeS@SC was investigated. The used adsorbents were collected by a magnet and washed with absolute alcohol to remove the ST before reused in the next adsorption experiment. The adsorption procedures of recycling experiments were the same as the first adsorption experiment, and the removal efficiencies of ST by the recycled adsorbents after different cycles were measured and calculated.

## 3. Results and Discussion

### 3.1. Characterization of the Adsorbents

#### 3.1.1. XRD Analysis

**Figure 2:**
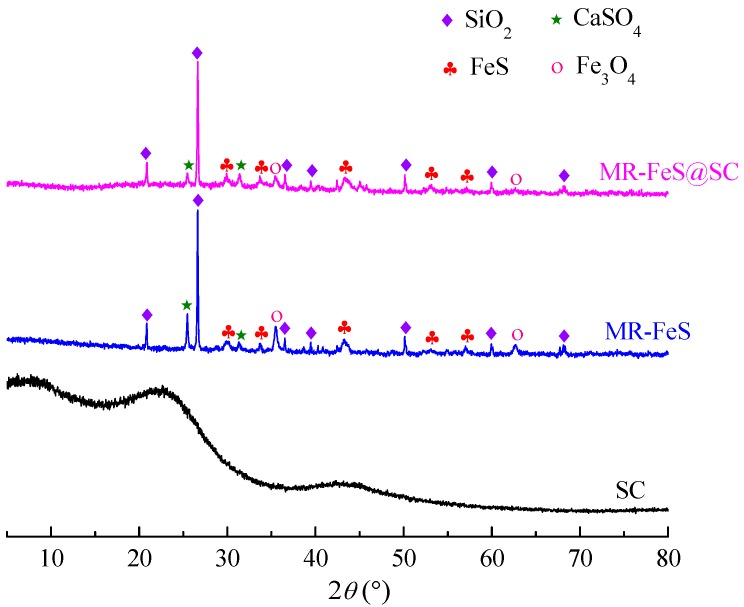
XRD patterns of SC, MR–FeS, and MR–FeS@SC.

The crystal structure of the samples was investigated by XRD analysis, and the XRD patterns of SC, MR–FeS, and MR–FeS@SC are presented in Figure 2. The pattern of SC shows broad peaks at approximately 20–26° and 42–45°, which are indexed to (002) and (101) diffraction planes of disordered carbon, respectively, indicating that the SC were mainly amorphous and had a highly disordered structure [21,29]. The XRD patterns of MR–FeS and MR–FeS@SC exhibit several sharp diffraction peaks, which are mainly different from those presenting in the raw manganese residue. The main crystalline phases were identified as FeS, SiO_2_, Fe_3_O_4_, and CaSO_4_, indicating that the phase transition occurred in the calcination process of manganese residue. FeS was successfully formed with or without the addition of starch. In addition, Fe_3_O_4_ was also formed during the calcination, which could impart magnetic properties to the adsorbents for easy separation from the solution.

#### 3.1.2. FESEM Analysis

**Figure 3:**
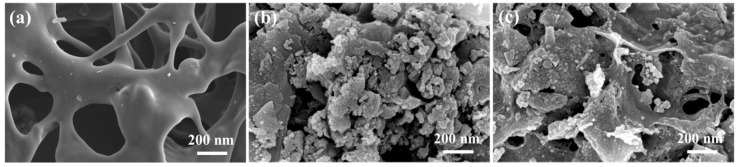
Field emission scanning electron microscopy (FESEM) images of (**a**) SC, (**b**) MR–FeS, and (**c**) MR–FeS@SC.

The surface morphologies of different samples were studied by field emission scanning electron microscopy (FESEM), and the images are shown in Figure 3. SC exhibited smooth surface with many round macropores, demonstrating that starch can be used to prepare porous carbon materials. The porous structure of SC mainly resulted from the expanded starch [25]. MR–FeS showed the aggregation of the particles with different sizes, from nanometer to micron scale. By comparison, the MR–FeS@SC nanocomposite displayed porous morphology with uniform distribution and embedding of inorganic nanoparticles in the SC, indicating that the addition of starch effectively reduced the agglomeration of these nanoparticles. This could be ascribed to the good dispersion of manganese residue in starch gel, and these nanoparticles were immobilized in SC by in-situ formation. Meanwhile, the uniform distribution of inorganic nanoparticles (especially FeS nanoparticles) in SC could help to increase the activities and adsorption properties of the nanocomposites.

#### 3.1.3. Specific Surface Area and Pore Size Analysis

**Figure 4:**
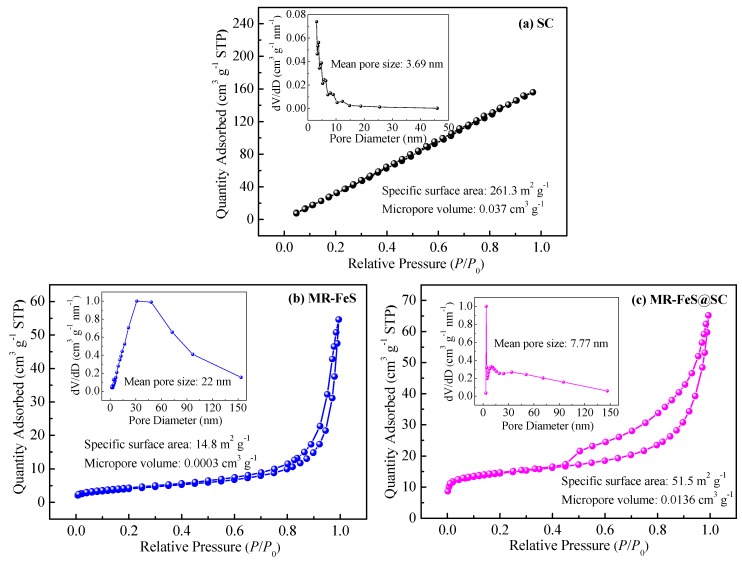
N_2_ adsorption–desorption isotherms and pore distributions of (**a**) SC, (**b**) MR–FeS, and (**c**) MR–FeS@SC.

N_2_ adsorption–desorption method was applied to determine the specific surface areas and pore size distributions of the samples, and the results are shown in Figure 4. Specific surface area, micropore volume, and pore size are important parameters for evaluating the adsorption properties of the adsorbents. Large specific surface area and pore volume and appropriate pore size can provide high adsorption capacity. The molecular size of ST is 1.14 nm × 0.92 nm, indicating that microporous adsorbent was better suitable for the adsorption of ST, thus micropore volume could mainly affect the adsorption capacity of the adsorbent [1,23]. Due to the low density and porosity of pure carbon material, SC exhibited larger specific surface area and micropore volume and smaller pore size compared with other two samples. MR–FeS possessed small specific surface area, very small micropore volume, and large pore size, which could not provide favorable adsorption conditions. As the good dispersion of MR–FeS nanoparticles in porous SC support, MR–FeS@SC nanocomposite showed relatively large specific surface area and micropore volume and appropriate pore size, which could exhibit better adsorption properties.

#### 3.1.4. FTIR Analysis

**Figure 5:**
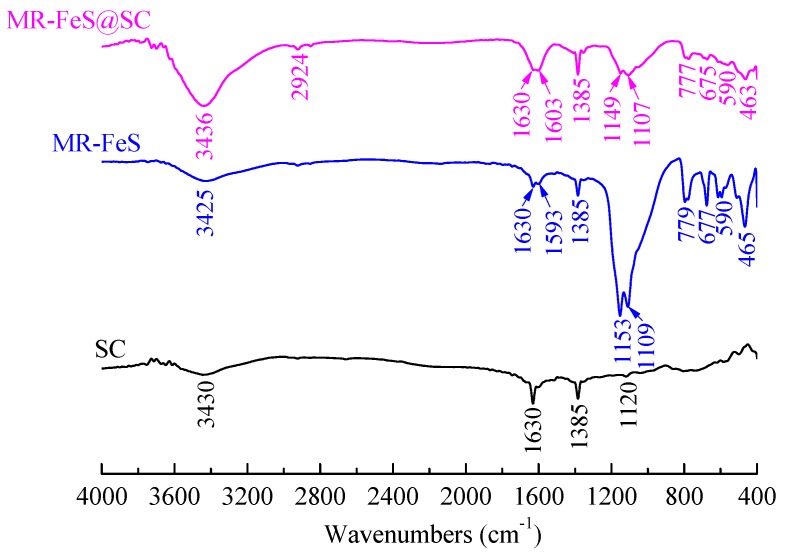
FTIR spectra of SC, MR–FeS, and MR–FeS@SC.

FTIR analysis was performed to qualitatively investigate the chemical functionalities of different samples, and the spectra are shown in Figure 5. In the spectrum of SC, a strong broad band centered at 3430 cm^−1^, and the peaks at 1630, 1385, and 1120 cm^−1^ were assigned to stretching vibration of –OH groups, stretching vibration of C=O, C–H bending, and stretching vibration of S=O (–SO_3_H), respectively [1,30,31,32], which were originated from the characteristics of starch and p-toluenesulfonic acid. Without the interaction of other substances, SC prepared by the carbonization of pure starch gel lost a large number of functional groups. In the spectrum of MR–FeS, the bands at 3425, 1630 and 1593, and 1385 cm^−1^ were assigned to stretching vibration of –OH groups, stretching vibration of C=O, and C–H bending, respectively, which were originated from the introduction of p-toluenesulfonic acid. The peaks at 1153 and 1109 cm^−^^1^ were attributed to the asymmetric absorption vibration of Si–O–Si and antisymmetric bridge stretching of C–O [1], and the peaks at 779, 677, and 465 cm^−^^1^ were attributed to bending vibration of Si–O, which confirmed the presence of SiO_2_ in MR–FeS. A peak at 590 cm^−1^ corresponded to the essential characteristic adsorption peak of Fe_3_O_4_ [33]. The spectrum of MR–FeS@SC nanocomposite shows the characteristic adsorption peaks as those in the spectra of SC and MR–FeS. With the interaction of manganese residue, the MR–FeS@SC prepared from the carbonization of the mixture of manganese residue and starch could retain more functional groups of starch. Moreover, the shift of these characteristic adsorption peaks in the spectrum of MR–FeS@SC compared with those in the spectra of SC and MR–FeS could be observed. A blue shift of the stretching vibration of –OH groups at 3436 cm^−^^1^ in the spectrum of MR–FeS@SC from 3430 cm^−^^1^ in SC and 3425 cm^−^^1^ in MR–FeS indicated the interaction between SC and MR–FeS through hydrogen bonds [34]. A blue shift of the stretching vibration of C=O at 1630 and 1603 cm^−^^1^ in the spectrum of MR–FeS@SC from 1630 and 1593 cm^−^^1^ in that of MR–FeS could also be observed, implying that the stable combination between SC and MR–FeS though the action of C=O. In addition, red shifts of the characteristic peaks of Si–O and C–O at 1149, 1107, 777, 675, and 463 cm^−^^1^ in the spectrum of MR–FeS@SC from 1153, 1109, 779, 677, and 465 cm^−^^1^ in that of MR–FeS indicated the presence of steric hindrance for combining SC and FeS though Si–O and C–O bonds. As the stable combination between SC and MR–FeS, the characteristic peak of Fe_3_O_4_ became weaker in the spectrum of MR–FeS@SC. FTIR analysis confirmed that MR–FeS nanoparticles were firmly combined with SC in the MR–FeS@SC nanocomposite though the bonding force of oxygen-containing groups, including –OH, C=O, C–O, Si–O, S=O, etc. The abundant functional groups in MR–FeS@SC nanocomposite are also beneficial to the improvement of its adsorption performance.

#### 3.1.5. Magnetic Behaviors

**Figure 6:**
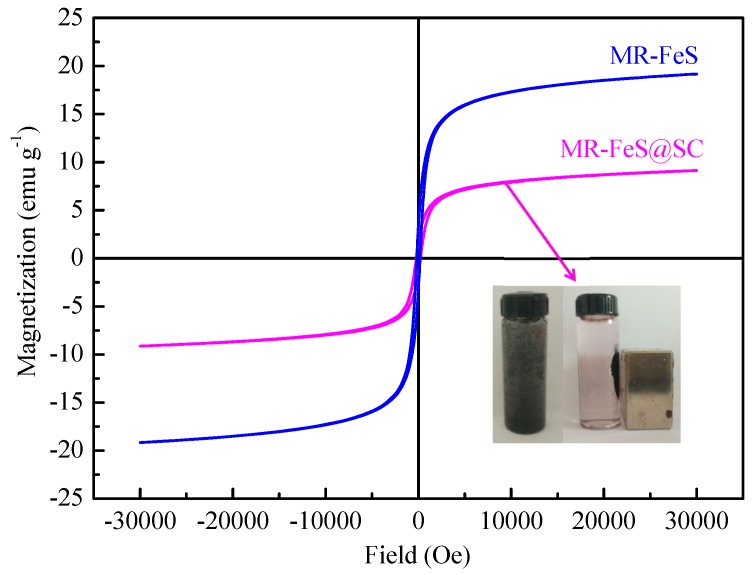
Hysteresis loops of MR–FeS and MR–FeS@SC (inset picture shows the separation of MR–FeS@SC from the aqueous solution by a magnet).

The magnetic properties of MR–FeS and MR–FeS@SC were examined by VSM at room temperature. Figure 6 shows that the saturation magnetization values for MR–FeS and MR–FeS@SC were determined to be 19.17 and 9.13 emu∙g^−1^, respectively. The saturation magnetization of MR–FeS@SC was lower than that of MR–FeS, probably owing to that SC was a nonmagnetic material and thus reduced the saturation magnetization of the nanocomposite. The reduction of saturation magnetization did not affect the separation of MR–FeS@SC from the aqueous solution system as the nanocomposite still exhibited superparamagnetic properties. The inset picture in Figure 6 shows that MR–FeS@SC nanocomposite was quickly separated from the aqueous solution by a magnet (external magnetic field) after becoming adsorption-saturated in the ST solution. The magnetic responsivity would be beneficial to the reuse of the adsorbents for applying in wastewater treatment [35].

#### 3.1.6. XPS Analysis

**Figure 7:**
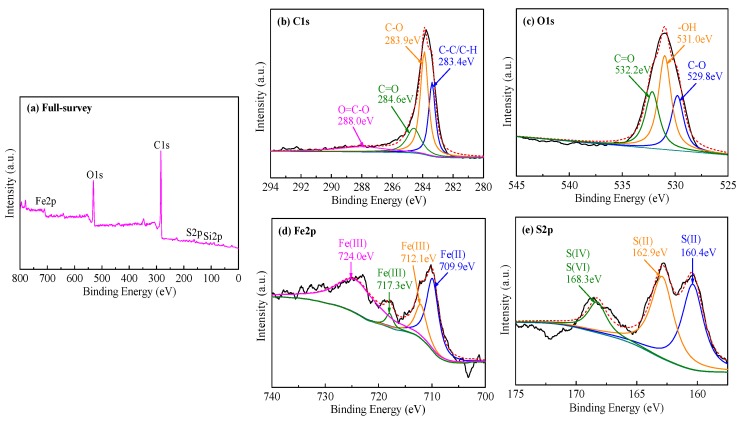
XPS spectra of MR–FeS@SC nanocomposite: (**a**) Full-survey spectrum and peak fitting curves of (**b**) C 1s, (**c**) O 1s, (**d**) Fe 2p, and (**e**) S 2p spectra.

The stoichiometric composition and chemical states of MR–FeS@SC nanocomposite were measured by XPS technique, and the results are presented in Figure 7. The full-survey spectrum (Figure 7a) shows that the main elements were C, O, Fe, Si, and S. The high-resolution C 1s, O 1s, Fe 2p, and S 2p XPS spectra provided detailed information referring to the surface chemistry. The C 1s spectrum was deconvoluted into four peaks at approximately 283.4, 283.9, 284.6, and 288.0 eV (Figure 7b), which were assigned to C–C/C–H, C–O, C=O, and O=C–O, respectively [29,36]. The O 1s core-level peak could be fitted into three peaks at 529.8, 531.0, and 532.2 eV (Figure 7c), ascribed to C–O, –OH, and C=O, respectively [12]. As shown in Figure 7d, the high-resolution Fe 2p spectrum was deconvoluted into four peaks. The binding energy at around 709.9 eV was the characteristic peak of Fe(II); the binding energies at approximately 712.1 and 717.3 eV were the characteristic peaks of Fe(III); the binding energies at around 724.0 eV was the characteristic peak of Fe(III)–O [37]. These characteristic peaks were mainly originated from FeS and Fe_3_O_4_, which is consistent with the XRD result. Figure 7e illustrates the high-resolution S 2p spectrum, which was deconvoluted into three peaks. The binding energies at approximately 160.4 and 162.9 eV were the characteristic peaks of S(II), ascribed to FeS; the binding energy at 168.3 eV was the characteristic peak of S(Ⅳ) and S(Ⅵ), ascribed to O–S–O and SO_4_^2−^ (CaSO_4_). Therefore, these XPS results are in good agreement with the results of FTIR and XRD.

### 3.2. Adsorption of ST by the Prepared Adsorbents

#### 3.2.1. Effect of Temperature

**Figure 8:**
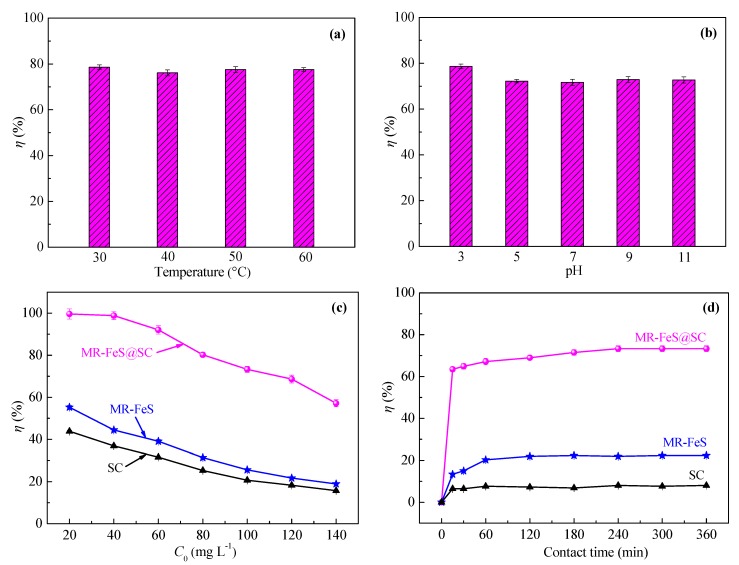
(**a**) Effect of temperature on the removal efficiency of Safranine T (ST) by MR–FeS@SC (adsorbent dosage = 0.8 g L^−1^, *C*_0_ (ST) = 100 g L^−1^, pH = 7.0, and contact time = 360 min); (**b**) effect of pH on the removal efficiency of ST by MR–FeS@SC (adsorbent dosage = 0.8 g L^−1^, *C*_0_ (ST) = 100 g L^−1^, temperature = 30 °C, and contact time = 360 min); (**c**) effect of initial ST concentration on the removal efficiency of ST by different adsorbents (adsorbent dosage = 0.8 g L^−1^, temperature = 30 °C, pH = 7.0, and contact time = 360 min); and (**d**) effect of contact time on the removal efficiency of ST by different adsorbents (adsorbent dosage = 0.8 g L^−1^, *C*_0_ (ST) = 100 g L^−1^, temperature = 30 °C, and pH = 7.0).

Commonly, temperature is considered as one of the main factors for affecting the adsorption process. The increase in temperature can lead to the decrease in the viscosity of the solution, which thus increases the diffusion rate of the adsorbate molecules across the external boundary layer and in the internal pores of the adsorbent particles [38]. Moreover, the equilibrium capacity of the adsorbent can be changed at different temperature for a particular adsorbate [10]. Herein, MR–FeS@SC nanocomposite was used to investigate the effect of temperature on the removal efficiency of ST, and the result is presented in Figure 8a, which shows that temperature did not have obvious effect on the adsorption capacity of MR–FeS@SC nanocomposite. This may be due to that the increased temperature was helpful to promote the diffusion of adsorbate into the adsorbent, but the adsorbate molecules were easy to be desorbed simultaneously. As a result, changing the temperature in a certain range had little effect on the adsorption process. It is optimum to operate the adsorption at room temperature (30 °C).

#### 3.2.2. Effect of pH

The pH of the solution can affect the surface charge of the adsorbents though the protonation or deprotonation of the functional groups, so pH is also considered to have a significant effect on the adsorption process [3]. Figure 8b illustrates the effect of the initial pH of ST solution on the adsorption capacity of MR–FeS@SC nanocomposite. It is clear that the adsorption of ST onto MR–FeS@SC nanocomposite was not remarkably dependent on the pH of the solution. MR–FeS@SC nanocomposite contained a variety of functional groups, which could play a role in the adsorption of ST under different pH conditions. Therefore, MR–FeS@SC nanocomposite adsorbent exhibited the characteristics of wide application range of pH and strong stability, and it could keep good adsorption performance under different conditions. Considering the practical applications, an optimal condition of pH = 7 was chosen for the following adsorption experiments.

#### 3.2.3. Effect of Initial Dye Concentration

Different initial concentration of adsorbate can provide different driving force in overcoming mass transfer resistance between the aqueous and the adsorbent. As shown in Figure 8c, the initial ST concentration had an important influence on the adsorption capacity of SC, MR–FeS, and MR–FeS@SC. The removal efficiency of ST decreased as the increase of ST concentration. When the initial ST concentration was low, small amount of ST molecules in the solution could be effectively adsorbed by the adsorbents with a large number of active sites on the surface. With the increase of initial ST concentration, the number of ST molecules in the solution increased, while the adsorption active sites on the surface of the adsorbents remained the same. As a result, the ST molecules were not completely adsorbed and remained in the solution, leading to the reduction in the removal efficiency of ST. It also can be observed that the order of adsorption capacity of the adsorbents was MR–FeS@SC ˃ MR–FeS ˃ SC. MR–FeS@SC nanocomposite was an efficient adsorbent for the removal of dye due to the relatively large specific surface area and micropore volume, appropriate pore size, plenty of various functional groups, and the interaction between the oxygen-containing functional groups of SC and MR–FeS nanoparticles.

#### 3.2.4. Effect of Contact Time

As an important parameter for adsorption, equilibrium time is commonly investigated. The influence of the contact time on the removal efficiency of ST using SC, MR–FeS, and MR–FeS@SC is presented in Figure 8d. It is clear to observe that the removal efficiencies were obviously faster at initial stage (0–15 min), especially for the adsorption process using MR–FeS@SC. The fast removal efficiency at the initial stage was mainly resulted from that all the active sites of the adsorbent were initially vacant, and the concentration gradient of the adsorbate molecules was high. The adsorption of ST onto SC, MR–FeS, and MR–FeS@SC reached complete equilibrium at approximately 120, 120, and 240 min, respectively. In addition, the removal efficiency of ST at equilibrium by MR–FeS@SC was much higher than that by SC or MR–FeS. The adsorption capacity of MR–FeS@SC was the best among these three absorbents, and that of SC was the worst, ascribed to the differences in their characteristics, including apparent structure, phase structure, functional groups, and the interaction between different components.

### 3.3. Adsorption Kinetics

**Figure 9:**
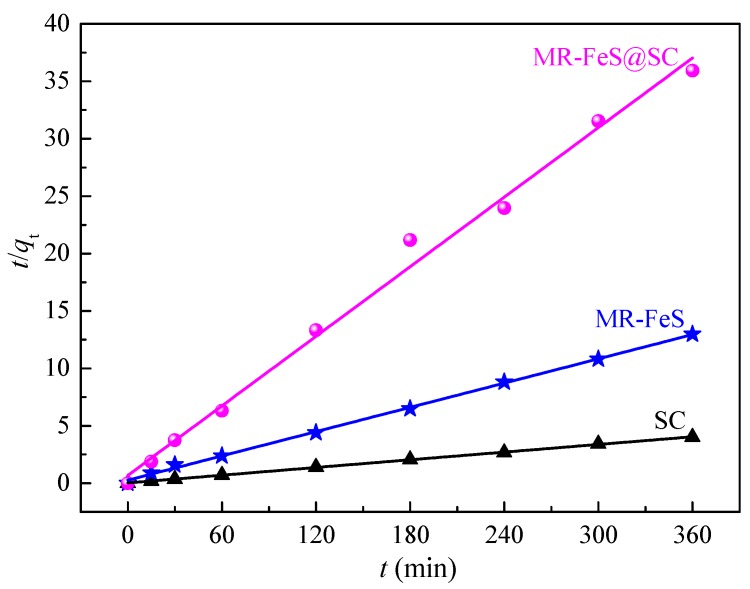
Pseudo-second-order kinetics for the adsorption of ST onto SC, MR–FeS, and MR–FeS@SC (adsorbent dosage = 0.8 g L^−1^, *C*_0_ (ST) = 100 g L^−1^, temperature = 30 °C, and pH = 7.0).

To understand the kinetic mechanism and the adsorption performances of different absorbents for ST, adsorption kinetics is necessary to be studied as it can provide valuable insights into both the adsorption mechanism and rate control steps of the adsorption process [39,40]. Based on the analyses of the characteristics of the adsorbents and the adsorption phenomena, pseudo-second-order kinetic model was employed to fit the experimental data. The pseudo-second-order model can be described using the following equation [41].

(3) $$\frac{t}{q_{t}}=\frac{1}{k_{2}q_{e}^{2}}+\frac{t}{q_{e}}$$

where *q*_e_ and *q*_t_ (mg g^−1^) are the adsorption capacities of the adsorbents for ST at equilibrium and at time *t*, respectively, *k*_2_ is the rate constant of pseudo-second-order (g mg^−1^ min^−1^). The plots of *t*/*q*_t_ versus *t* were depicted in Figure 9, which were used to calculate *k*_2_ values.

The parameters of the pseudo-second-order models for different adsorbents are shown in Table 2. The correlation coefficients (*R*^2^) values for the adsorption of ST onto SC, MR–FeS, and MR–FeS@SC were 0.9957, 0.9995, and 0.9996, respectively, indicating that the pseudo-second-order model fitted the experimental kinetic data well. In addition, the *q*_e_(cal) values calculated from the pseudo-second-order model were in good agreement with the experimental values *q*_e_(exp). These results demonstrate that the adsorption of ST by these three adsorbents was mainly chemisorption. It can be concluded that the rate step of adsorption was limited by chemical interaction, including valence forces involving exchanging or sharing electrons between the adsorbate and the adsorbent [41]. Moreover, the equilibrium adsorption capacity of MR–FeS@SC was more than three times that of MR–FeS and nine times that of SC, which were mainly attributed to the synergetic action between the functional groups of SC and MR–FeS nanoparticles.

### 3.4. Adsorption Isotherms

Commonly, adsorption isotherms are curves illustrating the relationship between the amount of the adsorbate that is adsorbed and the adsorbate concentration of the solution at equilibrium at a certain temperature and pH. Therefore, the strength of the interaction between adsorbent and adsorbate is evaluated [42]. Adsorption isotherms are obtained by theoretical models of adsorption, including theoretically assumed and mathematically deduced models. Certain isothermal parameters can be obtained after fitting the experimental data by the theoretical models. Several models have been demonstrated to be widely used for describing the experimental data of adsorption isotherms [41]. In this study, the Langmuir and Freundlich isotherms, two of the most commonly used models, were applied to fit with the experimental data to find the most suitable model [10].

**Figure 10:**
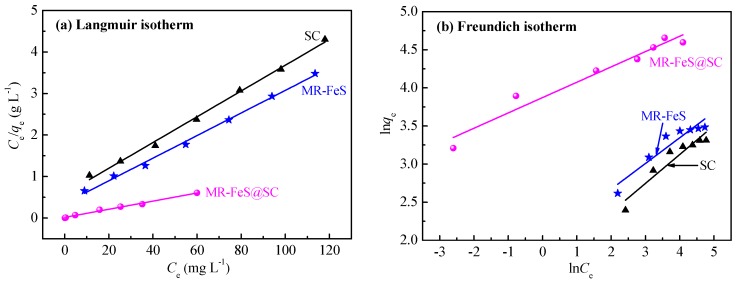
(**a**) Langmuir and (**b**) Freundlich isotherms for the adsorption of ST onto SC, MR–FeS, and MR–FeS@SC.

The expression of the Langmuir isotherm is shown as follows [43]

(4) $$\frac{C_{e}}{q_{e}}=\frac{C_{e}}{q_{m}}+\frac{1}{q_{m}k_{L}}$$

where *C*_e_ (mg L^−1^) is the concentration of the ST solution at equilibrium, *q*_e_ (mg g^−1^) is the adsorption capacity at equilibrium, *q*_m_ (mg g^−1^) is the maximum monolayer adsorption capacity, and *k*_L_ (L mg^−1^) is an adsorption equilibrium constant related to the adsorption capacity. As shown in Figure 10a, the plots of *C*_e_*/q*_e_ versus *C*_e_ are straight lines. The slopes and intercepts of the lines are equal to *1/q*_m_ and to *1/q*_m_*k*_L_, respectively.

The Freundlich isotherm model can be expressed as the following equation.

(5) $$\ln q_{e}=\ln k_{F}+\frac{1}{n}\ln C_{e}$$

where *C*_e_ and *q*_e_ are same to those in the Langmuir isotherm model and *k*_F_ (mg g^−1^) and 1/*n* are Freundlich adsorption constants related to adsorption capacity and adsorption intensity of the adsorbents [10]. The strength of the interaction between adsorbent and adsorbate can be explained using *k*_F_. The larger *k*_F_ value indicates the stronger adsorption of the adsorbent and the higher adsorption capacity (*q*_e_). The effect of the concentration of the solution during adsorption equilibrium can be explained by 1/*n* value. 0 < 1/*n* < 1 indicates that the adsorption is easily occurred with favorable adsorption process; 1/*n* = 1 indicates a linear adsorption process without any interactions between adsorbates; 1/*n* > 1 indicates that the adsorption is not easily occurred with unfavorable adsorption process [41]. The plots of ln*q*_e_ versus ln*C*_e_ were depicted in Figure 10b, and the values of *k*_F_ and *n* were obtained from these plots.

As presented in Table 3, Langmuir model fitted better than Freundlich model for all these three adsorbents. The *R*^2^ values were all larger than 0.99 when using the Langmuir isotherm to fit the adsorption of ST onto SC, MR–FeS, and MR–FeS@SC, which suggests that these adsorption processes were in good agreement with the Langmuir isotherm model. Therefore, these adsorption processes were mainly monolayer chemical adsorption processes. Moreover, the maximum monolayer adsorption capacity of MR–FeS@SC for ST was approximately three times that of SC or MR–FeS. The *n* values were all larger than 1 for the Freundlich isotherm model, indicating that the adsorption processes of ST by these three adsorbents were favorable and heterogeneous [44].

### 3.5. Reusability Analysis

**Figure 11:**
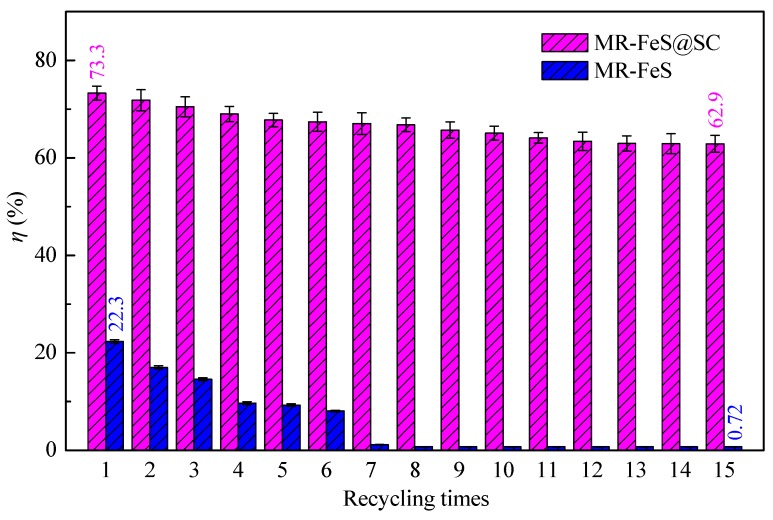
Removal efficiency of ST adsorbed by MR–FeS and MR–FeS@SC recycled for different times (adsorbent dosage = 0.8 g L^−1^, *C*_0_ (ST) = 100 g L^−1^, temperature = 30 °C, pH = 7.0, and contact time = 360 min).

The recyclability of adsorbent is very important to evaluate the economic feasibility and environmental friendliness for practical applications. The reusability of MR–FeS@SC nanocomposite was evaluated by conducting the adsorption–desorption process for fifteen cycles, and the reusability of MR–FeS was also conducted under the same conditions for comparative study. As presented in Figure 11, the removal efficiency of ST adsorbed by MR–FeS@SC nanocomposite slowly decreased as the increase of cycle times, but that by MR–FeS remarkably decreased, especially after six cycles. After fifteen cycles, the removal efficiency of ST adsorbed by MR–FeS@SC could be retained 62.9% (73.3% in the first cycle), but the MR–FeS recycled for over six times almost did not have adsorption capacity. These results confirm that the stability and recyclability of MR–FeS@SC nanocomposite were much higher than those of MR–FeS, which was mainly due to the strong interaction between MR–FeS and the functional groups of SC and the immobilization of MR–FeS nanoparticles onto SC support, reducing the loss of effective and active components. Consequently, the MR–FeS@SC nanocomposite could be considered an efficient and stable adsorbent for the removal of dyes from wastewater in practical applications.

## 4. Conclusions

In summary, a stable MR–FeS@SC nanocomposite was successfully prepared with manganese residue and starch as raw materials. With MR–FeS and SC as contrasts, the MR–FeS@SC adsorbent showed better adsorption properties for the removal of ST from the aqueous solution, attributed to relatively large specific surface area and micropore volume, appropriate pore size, plenty of functional groups, strong interaction between the functional groups of SC and MR–FeS, and the immobilization and uniform distribution of MR–FeS nanoparticles onto SC support. MR–FeS@SC and MR–FeS with superparamagnetic properties could be quickly separated from the solution using an external magnetic field. The adsorption could be conducted at a wide range of pH and temperature to achieve a satisfy removal efficiency of ST with MR–FeS@SC nanocomposite as adsorbent. The adsorption kinetics and isotherms indicated that the adsorption processes were mainly affected by chemisorption and were heterogeneous monolayer adsorption. Additionally, the MR–FeS@SC nanocomposite exhibited better reusability and stability even after fifteen cycles, attributed to the strong interaction between MR–FeS nanoparticles and SC support. Consequently, this novel approach for the preparation of low-cost adsorbent with natural material and industrial waste as raw materials is feasible and promising for treating organic pollutants-bearing wastewater in practical applications.

## Figures and Tables

**Table 1:** Major chemical compositions of the manganese residue.

Element	O	Fe	Si	S	Ca	Al	Mn	Ba	K
**Mass fraction (%)**	43.8	22.0	12.0	10.0	4.0	3.0	1.0	1.0	0.9

**Table 2:** Pseudo-second-order kinetic parameters for the adsorption of ST by different adsorbents.

Adsorbent	*q*_e_(cal) (mg g^−1^)	*q*_e_(exp) (mg g^−1^)	*k*_2_ (g mg^−1^ min^−1^)	*R* ^2^
SC	10.02	10.01	0.0098	0.9957
MR–FeS	27.82	28.04	0.0067	0.9995
MR–FeS@SC	92.34	93.52	0.0016	0.9996

**Table 3:** Langmuir and Freundlich isotherms parameters for the adsorption of ST by different adsorbents.

Adsorbent	Langmuir Isotherm			Freundlich Isotherm		
	*q*_m_ (mg g^−1^)	*k*_L_ (L mg^−1^)	*R* ^2^	*n*	*k*_F_ (L mg^−1^)	*R* ^2^
SC	32.22	0.054	0.9956	2.64	5.00	0.8873
MR–FeS	36.74	0.077	0.9960	2.96	7.36	0.8758
MR–FeS@SC	102.14	0.639	0.9918	4.97	48.06	0.9471
